# Author Correction: Modularity increases rate of floral evolution and adaptive success for functionally specialized pollination systems

**DOI:** 10.1038/s42003-019-0725-7

**Published:** 2019-12-12

**Authors:** Agnes S. Dellinger, Silvia Artuso, Susanne Pamperl, Fabián A. Michelangeli, Darin S. Penneys, Diana M. Fernández-Fernández, Marcela Alvear, Frank Almeda, W. Scott Armbruster, Yannick Staedler, Jürg Schönenberger

**Affiliations:** 1 Department of Botany and Biodiversity Research, University of Vienna, Rennweg 14, 1030 Vienna, Austria; 2 Department of Biosciences, University of Salzburg, Hellbrunnerstraße 34, 5020 Salzburg, Austria; 3 Institute of Systematic Botany, The New York Botanical Garden, 2900 Southern Blvd, Bronx, NY 10458-5126 USA; 4 Department of Biology and Marine Biology, University of North Carolina Wilmington, 601S. College Road, Wilmington, NC 28403 USA; 5 Herbario Nacional del Ecuador (QCNE), Instituto Nacional de Biodiversidad, Río Coca E06-115 e Isla Fernandina, Quito, Ecuador; 6 Institute of Biodiversity Science and Sustainability, California Academy of Sciences, 55 Music Concourse Drive, San Francisco, CA 94118-4503 USA; 7 School of Biological Science, University of Portsmouth, King Henry 1 Street, Portsmouth, P012DY UK

Correction to: *Communications Biology* 10.1038/s42003-019-0697-7, published online 5 December 2019.

The original version of this Article contained an error in the spelling of the author Yannick Staedler, which was incorrectly given as Yannick Staeder. This has now been corrected in both the PDF and HTML versions of the Article.

