# Supplementary material for: Modularity increases rate of floral evolution and adaptive success for functionally specialized pollination systems
Source: Commun Biol. 2019 Dec 5;2:453. doi: 10.1038/s42003-019-0697-7 (PMC6895197; doi:10.1038/s42003-019-0697-7)
Supplement: Supplementary file 1 — Supplementary Information [file 42003_2019_697_MOESM1_ESM.pdf]

Supplementary Figures

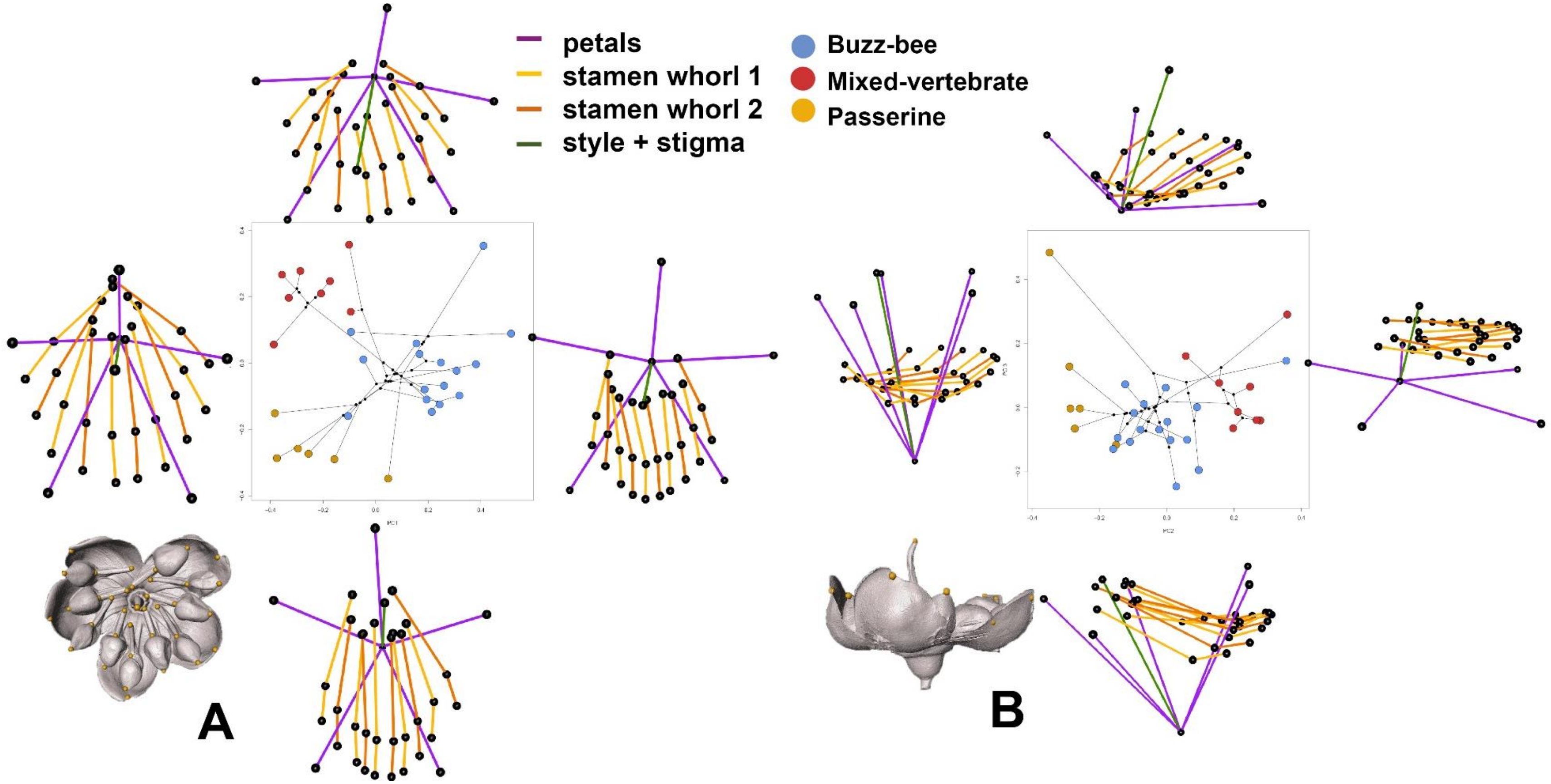

Supplementary Figure 1. Flower shape change (organs represented by wireframes) in the phylo-morphospace (Fig. 3D in main text). A) Shape change when flower is viewed from the front; note the change from a tightly aggregated, monosymmetric androecium at the positive extreme of PC1 and the negative extreme of PC2 ('buzz-bee' and 'mixed-vertebrate' flowers), while stamens are arranged more broadly at the negative extreme of PC1 and positive extreme of PC2. ('passerine' syndrome flowers). B) Shape change when flower is viewed from the side; marked change in corolla shape becomes apparent from reflexed/open corolla to pseudo-tubular corolla along PC1; also note change in androecial arrangement from bent in stamens (positive extreme of PC1) to

reflexed stamens with anther pores elevated towards the stigma (negative extreme of PC2). Colour coding of wireframes follows the developmental Hyp. 1 (Fig. 1 in main text).

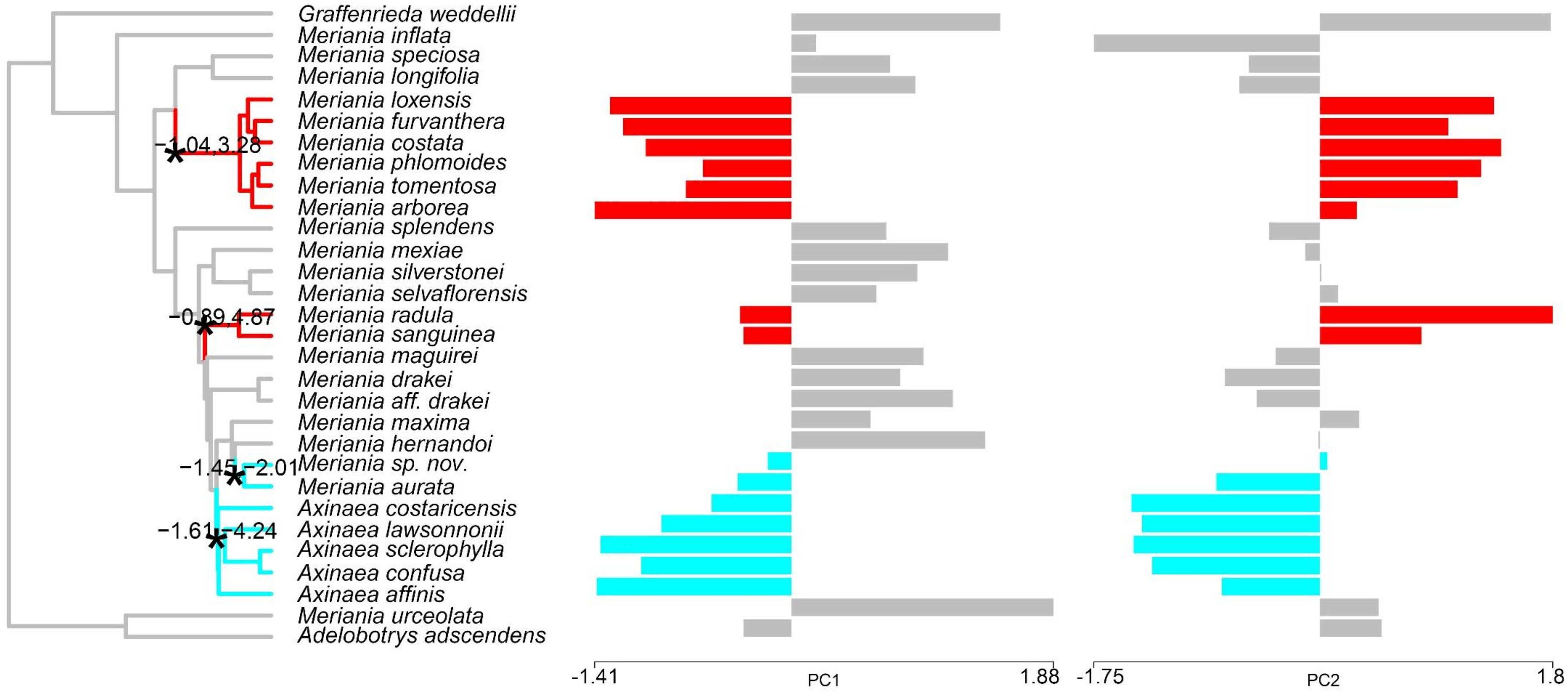

Supplementary Figure 2. The four estimated shifts in phenotypic optima assessed on PC1 and PC2 of Merianieae floral shape space on the MCC-tree. Significant shifts are indicated by asterisks and represent three of the four transitions from 'buzz-bee' to vertebrate pollination, and one transition within the 'buzz-bee' syndrome. The red colouration of two shifts indicates convergence in floral shape within the 'mixed-vertebrate' syndrome, the blue colouration indicates convergence in the shift to the 'passerine' syndrome and two 'buzz-bee' syndrome species with salverform corolla shapes. Regime shifts were evaluated on PC1 and PC2, PC1 summarizes corolla shape from reflexed (only 'buzz-bee') to pseudo-tubular corollas in the two vertebrate pollination syndromes and some 'buzz-bee' syndrome species. PC2 summarizes stamen arrangement, reflexed in the 'mixed-vertebrate' syndrome (and few 'buzz-bee' syndrome species) and bent-in in the 'passerine' and most 'buzz-bee' syndrome species.

Supplementary Tables

Supplementary Table 1. Specification of the five hypotheses of modularity, landmark partitioning, source of modularity hypothesis and expectations for the three different pollination syndromes in Merianieae.

Hypotheses	Landmark partitioning	Source	Expectations for pollination syndromes
Hyp. 1: *Developmental units*: corolla (M1), outer stamen whorl (M2), inner stamen whorl (M3), style (M4)	M1: 33-37; M2: 1,3,5,7,9,11,13, 15,17,19,21,23,25,27,29; M3: 2,4,6,8,10,12,14,16,18,20,22,24,26,28,30; M4: 31,32	1	no expectation
Hyp. 2: *Attraction* (M1) versus *reproductive organs* (M2)	M1: 31, 33-37<br>M2: 1-30, 32	1; 2	*modularity of corolla as an attraction trait in all systems*
Hyp. 3: Attraction (*corolla and stamen appendages*, M1) versus efficiency (*pore locations and stigma,* M2)	M1: 1-20, 31, 33-37<br>M2: 21-30, 32	3	*modularity in 'buzz-bee' syndrome* (corolla as landing platform and stamens as handles for buzzing) but not in 'mixed-vertebrate' and 'passerine'
Hyp. 4: Attraction *(appendages,* M1) versus efficiency (*pore location, stigma and corolla*, M2)	M1: 1-20,<br>M2: 21-37	3	*modularity in 'passerine' and possibly 'mixed-vertebrate' syndrome* (corolla mediates fit, appendages trigger bellows in 'passerine') but no fit-functoin in 'buzz-bee' syndrome
Hyp. 5: *Merianieae specific functional modules*: attraction *(corolla,* M1) versus pollen expulsion (*stamen appendages*, M2) versus efficiency (*pore locations and stigma, pollen transfer*, M3)	M1: 31, 33-37<br>M2: 1-20<br>M3: 21-30, 32	this study	*modularity in 'buzz-bee' and 'passerine' syndrome* (appendages as triggers for pollen expulsion mechanisms) but not 'mixed-vertebrate' syndrome

Supplementary Results

Supplementary Table 2. Pairwise comparison of effect sizes of the five hypotheses on floral modularity for the three different pollination syndromes. The lower off-diagonal values represent the pairwise differences in z-scores (effect sizes) of the CR coefficient, the upper off-diagonal gives their associated p-values (i.e. p<0.05 indicating a significant difference in modularity; significant p-values printed in italics); n = 137 specimens.

**Hyp. 1**	**buzz-bee**	**mixed-vertebrate**	**passerine**
**buzz-bee**		*0.013*	*0.033*
**mixed-vertebrate**	2.223		0.343
**passerine**	1.842	0.403	
**Hyp. 2**	**buzz-bee**	**mixed-vertebrate**	**passerine**
**buzz-bee**		*0.050*	*0.048*
**mixed-vertebrate**	1.640		0.492
**passerine**	1.666	0.020	
**Hyp. 3**	**buzz-bee**	**mixed-vertebrate**	**passerine**
**buzz-bee**		0.111	0.080
**mixed-vertebrate**	1.223		0.464
**passerine**	1.403	0.090	
**Hyp. 4**	**buzz-bee**	**mixed-vertebrate**	**passerine**
**buzz-bee**		*< 0.001*	0.130
**mixed-vertebrate**	3.299		*< 0.001*
**passerine**	1.129	3.567	
**Hyp. 5**	**buzz-bee**	**mixed-vertebrate**	**passerine**
**buzz-bee**		*0.009*	0.065
**mixed-vertebrate**	2.381		0.058
**passerine**	1.514	1.570	

Supplementary Table 3. Results from the rarefaction analyses of the five different hypotheses on modularity (Fig. 2) for the three pollination syndromes. The averages over 100 runs are given (n = 100). Highest modularity was found in the 'buzz-bee' syndrome and lowest modularity in the 'mixed-vertebrate' syndrome. In contrast to analyses on the mean shape, evolutionary modularity accounting for phylogenetic relatedness (column "Merianieae") was only found in Hyp. 2 in the resampled dataset. CR – Covariance Ratio. p – p-value <0.05 indicates significantly smaller CR than expected when no modularity is present. Z – effect sizes of CR.

**modularity hypothesis**	**buzz-bee**			**mixed-vertebrate**			**passerine**			**Merianieae**	
	**CR**	**p**	**Z**	**CR**	**p**	**Z**	**CR**	**p**	**Z**	**CR**	**p**
**Hyp. 1**	0.815	*0.001*	2.522	0.109	0.706	0.831	1.055	0.335	0.369	1.547	0.998

**Hyp. 2**	0.885	*0.037*	1.910	1.005	0.328	0.547	1.012	0.195	0.640	0.994	*0.256*
**Hyp. 3**	0.943	0.056	1.915	0.979	0.199	1.087	0.996	0.114	1.406	0.949	0.087
**Hyp. 4**	0.812	*0.001*	5.722	0.925	0.058	2.609	0.814	*0.001*	11.149	1.018	0.355
**Hyp. 5**	0.839	*0.002*	3.303	0.954	0.074	1.852	0.903	*0.005*	4.175	0.97	0.118

Supplementary Table 4. Results from the down sampling analyses (50%) of the five different hypotheses on modularity for the three pollination syndromes. The averages over 100 runs are given (n = 100). Congruent with the analyses on the full dataset, highest modularity was found in the 'buzz-bee' syndrome and lowest modularity in the 'mixed-vertebrate' syndrome. In contrast to the full dataset, there was no significant modularity in the 'mixed-vertebrate' syndrome. CR – Covariance Ratio. p – p-value <0.05 indicates significantly smaller CR than expected when no modularity is present. Z – effect sizes of CR.

**modularity**	**buzz-bee**			**mixed-vertebrate**			**passerine**		
**hypothesis**	**CR**	**p**	**Z**	**CR**	**p**	**Z**	**CR**	**p**	**Z**
**Hyp. 1**	0.934	*0.050*	1.910	1.138	0.617	1.036	1.056	0.301	0.697
**Hyp. 2**	0.937	*0.061*	1.776	1.015	0.307	0.916	1.027	0.273	0.697
**Hyp. 3**	0.968	0.082	1.807	1.001	0.261	1.219	0.985	0.137	2.285
**Hyp. 4**	0.898	*0.010*	4.459	0.990	0.179	1.758	0.908	*0.029*	7.984
**Hyp. 5**	0.911	*0.009*	2.963	1.002	0.147	1.501	0.963	*0.045*	3.015

Supplementary Table 5. The number of times a modularity hypothesis was found to be significant in the rarefied and the down sampled (to 50%) datasets over 100 runs each; n = 100.

**rarefaction**	**Hyp. 1**	**Hyp. 2**	**Hyp. 3**	**Hyp. 4**	**Hyp. 5**
**buzz-bee**	100	75	92	100	100
**mixed-vertebrate**	0	1	7	59	50
**passerine**	0	0	28	100	100
**Merianieae**	0	5	92	46	64

**down sampling**	**Hyp. 1**	**Hyp. 2**	**Hyp. 3**	**Hyp. 4**	**Hyp. 5**
**buzz-bee**	100	74	55	100	100
**mixed-vertebrate**	0	0	17	66	53
**passerine**	0	0	33	100	100

Supplementary Table 6. The number of times strength of modularity (calculated through z-scores) was significantly different between pollination syndromes. The upper off-diagonal gives results for the rarefied dataset, the lower off-diagonal gives results for the down sampled (to 50%) datasets over 100 runs each. Results were consistent between the two analyses, apart from Hyp. 3, where we detected higher modularity in the rarefied dataset than in the down sample dataset; n = 100.

**Hyp. 1**	**buzz-bee**	**mixed-vertebrate**	**passerine**
**buzz-bee**		*98*	*72*
**mixed-vertebrate**	96		6
**passerine**	90	5	

Hyp. 2	buzz-bee	mixed-vertebrate	passerine
buzz-bee		*25*	*16*
mixed-vertebrate	29		0
passerine	24	0	

Hyp. 3	buzz-bee	mixed-vertebrate	passerine
buzz-bee		43	48
mixed-vertebrate	8		0
passerine	20	1	

Hyp. 4	buzz-bee	mixed-vertebrate	passerine
buzz-bee		*90*	27
mixed-vertebrate	77		*90*
passerine	23	83	

Hyp. 5	buzz-bee	mixed-vertebrate	passerine
buzz-bee		*94*	29
mixed-vertebrate	74		41
passerine	23	36	

Supplementary Table 7. Model parameters and log-likelihood fits for the five hypotheses of modularity (Hyp1-5) and a hypothesis of no modularity for all Merianieae species of this study (n=30) and the different pollination syndromes separately. The optimal model for each dataset is highlighted in bold. Each modularity hypothesis is also analysed for within- and intermodule correlations: 'same.Mod'- similar correlations within the modules, 'same.between' – similar correlations between modules; 'sep.Mod' – different correlations within the modules, 'sep.between' – different correlations between modules (see 12 for explanation.

Merianieae	LogL	K	n	AICc	dAICc	Model LogL	Model Posterior Probability
**Null modularity**	693.3	3	666	-1380.7	83.5	0.000	0.000
**Hyp1.same.Mod + same.between**	705.5	6	666	-1398.9	65.2	0.000	0.000
**Hyp1.sep.Mod + same.between**	700.5	8	666	-1384.7	79.4	0.000	0.000
**Hyp1.same.Mod + sep.between**	712.6	11	666	-1402.8	61.3	0.000	0.000
**Hyp1.sep.Mod + sep.between**	689.7	2	666	-1375.4	88.7	0.000	0.000
**Hyp2.same.Mod + same.between**	691.7	3	666	-1377.4	86.7	0.000	0.000
**Hyp2.sep.Mod + same.between**	699.6	4	666	-1391.1	73.0	0.000	0.000
**Hyp3.same.Mod + same.between**	725.5	3	666	-1444.9	19.2	0.000	0.000
**Hyp3.sep.Mod + same.between**	728.5	4	666	-1448.9	15.3	0.000	0.000
**Hyp4.same.Mod + same.between**	715.5	3	666	-1425.0	39.1	0.000	0.000
**Hyp4.sep.Mod + same.between**	716.1	4	666	-1424.1	40.0	0.000	0.000
**Hyp5.same.Mod + same.between**	729.2	3	666	-1452.3	11.8	0.003	0.002
**Hyp5.sep.Mod + same.between**	733.8	5	666	-1457.6	6.6	0.038	0.034

**Hyp5.same.Mod + sep.between**	734.5	5	666	-1458.9	5.2	0.074	0.066
**Hyp5.sep.Mod + sep.between**	**739.1**	**7**	**666**	**-1464.1**	**0.0**	**1.000**	**0.897**
**buzz-bee' syndrome**							
**Null modularity**	645.1	2	666	-1286.1	26.5	0.000	0.000
**Hyp1.same.Mod + same.between**	648.9	3	666	-1291.8	20.9	0.000	0.000
**Hyp1.sep.Mod + same.between**	651.9	6	666	-1291.7	21.0	0.000	0.000
**Hyp1.same.Mod + sep.between**	654.8	8	666	-1293.4	19.3	0.000	0.000
**Hyp1.sep.Mod + sep.between**	657.8	11	666	-1293.2	19.5	0.000	0.000
**Hyp2.same.Mod + same.between**	647.0	3	666	-1288.0	24.6	0.000	0.000
**Hyp2.sep.Mod + same.between**	648.7	4	666	-1289.2	23.4	0.000	0.000
**Hyp3.same.Mod + same.between**	647.5	3	666	-1288.9	23.7	0.000	0.000
**Hyp3.sep.Mod + same.between**	651.0	4	666	-1293.9	18.8	0.000	0.000
**Hyp4.same.Mod + same.between**	**659.3**	**3**	**666**	**-1312.7**	**0.0**	**1.000**	**0.441**
**Hyp4.sep.Mod + same.between**	660.0	4	666	-1311.9	0.7	0.694	0.306
**Hyp5.same.Mod + same.between**	656.5	3	666	-1306.9	5.7	0.057	0.025
**Hyp5.sep.Mod + same.between**	658.4	5	666	-1306.7	6.0	0.051	0.022
**Hyp5.same.Mod + sep.between**	660.0	5	666	-1309.9	2.8	0.248	0.109
**Hyp5.sep.Mod + sep.between**	661.9	7	666	-1309.6	3.0	0.218	0.096
**mixed-vertebrate' syndrome**							
**Null modularity**	399.0	2	666	-793.9	7.8	0.020	0.007
**Hyp1.same.Mod + same.between**	399.0	3	666	-791.9	9.8	0.008	0.003
**Hyp1.sep.Mod + same.between**	399.6	6	666	-787.1	14.6	0.001	0.000
**Hyp1.same.Mod + sep.between**	402.1	8	666	-788.0	13.7	0.001	0.000
**Hyp1.sep.Mod + sep.between**	402.7	11	666	-783.1	18.6	0.000	0.000
**Hyp2.same.Mod + same.between**	400.5	3	666	-795.0	6.8	0.034	0.012
**Hyp2.sep.Mod + same.between**	400.5	4	666	-793.0	8.7	0.013	0.004
**Hyp3.same.Mod + same.between**	399.7	3	666	-793.4	8.3	0.016	0.006
**Hyp3.sep.Mod + same.between**	401.3	4	666	-794.5	7.2	0.028	0.010
**Hyp4.same.Mod + same.between**	**403.9**	**3**	**666**	**-801.7**	**0.0**	**1.000**	**0.353**
**Hyp4.sep.Mod + same.between**	403.9	4	666	-799.7	2.0	0.363	0.128
**Hyp5.same.Mod + same.between**	403.7	3	666	-801.3	0.4	0.808	0.285
**Hyp5.sep.Mod + same.between**	404.2	5	666	-798.3	3.4	0.186	0.066
**Hyp5.same.Mod + sep.between**	404.7	5	666	-799.2	2.5	0.291	0.103
**Hyp5.sep.Mod + sep.between**	405.2	7	666	-796.3	5.4	0.066	0.023
**passerine-syndrome**							
**Null modularity**	294.0	2	666	-584.0	7.4	0.025	0.011
**Hyp1.same.Mod + same.between**	294.2	3	666	-582.3	9.1	0.011	0.005
**Hyp1.sep.Mod + same.between**	296.4	6	666	-580.6	10.8	0.005	0.002
**Hyp1.same.Mod + sep.between**	294.5	8	666	-572.9	18.5	0.000	0.000
**Hyp1.sep.Mod + sep.between**	296.7	11	666	-571.1	20.3	0.000	0.000
**Hyp2.same.Mod + same.between**	294.4	3	666	-582.7	8.7	0.013	0.006
**Hyp2.sep.Mod + same.between**	296.8	4	666	-585.5	5.9	0.052	0.023
**Hyp3.same.Mod + same.between**	294.5	3	666	-583.0	8.4	0.015	0.007
**Hyp3.sep.Mod + same.between**	294.9	4	666	-581.7	9.7	0.008	0.003

**Hyp4.same.Mod + same.between**	**298.7**	**3**	**666**	**-591.4**	**0.0**	**1.000**	**0.437**
**Hyp4.sep.Mod + same.between**	299.2	4	666	-590.3	1.1	0.565	0.247
**Hyp5.same.Mod + same.between**	297.1	3	666	-588.1	3.3	0.190	0.083
**Hyp5.sep.Mod + same.between**	298.8	5	666	-587.5	3.9	0.145	0.063
**Hyp5.same.Mod + sep.between**	298.8	5	666	-587.6	3.8	0.148	0.065
**Hyp5.sep.Mod + sep.between**	300.6	7	666	-587.0	4.4	0.111	0.049

K – Model parameters. LogL – raw log-likelihood fits for each model. AICc – finite sample corrected Akaike Information Criterion. dAICc – difference between lowest AICc and each respective AICc. Model LogL – Model log-likelihood.

Supplementary Table 8. Summary table of log-likelihood ratio tests on model fit on the rarefied and down sampled (to 50%) datasets. The number of times any of the five hypotheses of modularity or a null-hypothesis of no modularity was found as best fit is given (100 runs; n = 100).

rarefaction	Hyp. 1	Hyp. 2	Hyp. 3	Hyp. 4	Hyp. 5	no modularity
**buzz-bee**	1	0	0	88	6	0
**mixed-vertebrate**	0	0	0	54	46	0
**passerine**	0	0	0	100	0	0
**Merianieae**	0	0	0	0	100	0

down sampling	Hyp. 1	Hyp. 2	Hyp. 3	Hyp. 4	Hyp. 5	no modularity
**buzz-bee**	11	1	3	63	22	0
**mixed-vertebrate**	0	1	5	40	10	44
**passerine**	0	1	0	53	10	36

Supplementary Table 9. Summary table of rarefaction analysis on differences in evolutionary rate of the different functional modules defined by Hyp. 4 and Hyp. 5. Averages over 100 runs are given, * indicate that significant differences were detected in all pairwise comparisons; these significant differences were detected in all 100 rarefaction runs and corolla shape always evolved significantly faster than the rest of the flower; n = 100.

**Hyp. 4**	***R = 3.46, p = 0.001***
attraction	0.00018*
efficiency	0.00060*

**Hyp. 5**	***R = 5.34, p = 0.001***
attraction (corolla)	0.00093*
pollen expulsion	0.00018*
efficiency	0.00043*

Supplementary Table 10. Comparison of fit of four different models of trait evolution on landmark data as assessed by GIC; estimated parameter values are given. The average GIC over 100 rarefaction runs is given. Note that the analysis of the original dataset resolved the OU-model as best fit while in the rarefaction analyses, the lambda model was found to fit the data best in 100% of cases.

model	GIC	parameter	average GIC
Brownian motion	-15‘370	-	-14‘033

lambda	-16‘477	lambda 1e-5	-14‘724
early burst	-15‘368	r 0	-14‘031
**Ornstein-Uhlenbeck**	**-16‘518**	**alpha 10**	-14‘500

Supplementary Table 11. Proportion of times a species was included in a regime shift for the resampled trait dataset. Note that with one exception (*M. inflata*) all species which have shifted pollination syndrome (‘mixed-vertebrate’ or ‘passerine’) were also found to have undergone a shift in phenotypic optimum as measured by PC1 and PC2; n = 30, 100 resampling runs.

species	% shifts	pollination syndrome
Axinaea affinis	103	passerine
Axinaea cf. lawessonii	103	passerine
Axinaea confusa	103	passerine
Axinaea sclerophylla	103	passerine
Axinaea costaricensis	94	passerine
Meriania phlomoides	78	mixed-vertebrate
Meriania arborea	70	mixed-vertebrate
Meriania tomentosa	69	mixed-vertebrate
Meriania loxensis	66	mixed-vertebrate
Meriania costata	66	mixed-vertebrate
Meriania furvanthera	66	mixed-vertebrate
Meriania radula	58	mixed-vertebrate
Meriania sanguinea	54	mixed-vertebrate
Meriania aurata	35	buzz-bee
Meriania sp. nov	35	buzz-bee
Meriania hernandoi	19	buzz-bee
Meriania maxima	9	buzz-bee
Meriania drakei	7	buzz-bee
Graffenrieda weddellii	5	buzz-bee
Adelobotrys adscendens	5	buzz-bee
Meriania aff. drakei	4	buzz-bee
Meriania inflata	2	passerine
Meriania longifolia	2	buzz-bee
Meriania maguirei	2	buzz-bee
Meriania mexiae	2	buzz-bee
Meriania selvaflorensis	2	buzz-bee
Meriania silverstonei	2	buzz-bee
Meriania speciosa	2	buzz-bee
Meriania splendens	2	buzz-bee
Meriania urceolata	0	buzz-bee

Supplementary Table 12. Comparison of fit of the two different OU-models on PC1 and PC2 on shape means and best fit model for randomly rarefied trait datasets (% of best fit from 100 runs, n = 100).

model	pBIC	difference pBIC	w	% best fit model

estimated shifts	-31.38	9.779	0.008	36
**estimated convergence**	**-41.161**	**0.000**	**0.993**	**64**

Supplementary Methods

Supplementary Table 13. Taxon sampling; details on sampling localities, voucher information, number of flowers used for HRX-CT scanning from each locality and pollination syndromes ('buzz-bee', 'mixed vertebrate', 'passerine') are given. For species with known pollinators, references for pollinator observations are given in brackets, for species with unknown pollinators, pollination syndrome classifications have been adopted from Dellinger et al. (4).

Species	Collection number	Collector	Country	State/Province	Collection date	Voucher	No. of flowers	Pollinator
Adelobotrys adscendens (Sw.) Triana	FA10230	Frank Almeda	Colombia	Valle del Cauca	04.02.2011	CAS 1120080	9	buzz-bee (4)
Adelobotrys adscendens (Sw.) Triana	RK5676	Ricardo Kriebel	Panamá	PN Chagres	14.09.2011	NYBG1653386	2	
Axinaea affinis (Naudin) Cogn.	AD41	Agnes Dellinger	Ecuador	Azuay	29.11.2012	-	1	passerine
Axinaea cf. *lawessonii* E. Cotton	AD112	Agnes Dellinger	Ecuador	Loja, PN Podocarpus	11.09.2016	WU 0092822	1	passerine
Axinaea confusa E. Cotton & Borchs.	AD136	Agnes Dellinger	Ecuador	Loja, PN Podocarpus	18.09.2016	WU 0092808	10	passerine (4)
Axinaea costaricensis Cogn.	AD75	Agnes Dellinger	Costa Rica	San José, highway to Cerro de la Muerte	02.02.2015	WU	18	passerine (4)
Axinaea costaricensis Cogn.	AD74	Agnes Dellinger	Costa Rica	San José, Cerros de Escazú	12.03.2015	WU	11	
Axinaea sclerophylla Triana	AD109	Agnes Dellinger	Ecuador	Loja, PN Podocarpus	10.09.2016	WU 0092819	1	passerine
Graffenrieda weddellii Naudin	MA1503	Marcela Alvear	Colombia	Risaralda	05.01.2014	CAS 1155724	1	buzz-bee
Meriania arborea Triana	FA10564	Frank Almeda	Colombia	Norte de Santander	28.02.2012	CAS 1128115	1	mixed-vertebrate
Meriania aff. drakei	AD140	Agnes Dellinger	Ecuador	Pastaza, Reserva EcoMinga	13.11.2016	QCNE	4	buzz-bee
Meriania aff. drakei	AD141	Agnes Dellinger	Ecuador	Pastaza, Reserva EcoMinga	13.11.2016	QCNE	3	buzz-bee
Meriania aff. drakei	AD141B	Agnes Dellinger	Ecuador	Pastaza, Reserva EcoMinga	13.11.2016	QCNE	2	buzz-bee

Meriania aff. drakei	AD152	Agnes Dellinger	Ecuador	Pastaza, Reserva EcoMinga	15.11.2016	WU 0092853	2	buzz-bee
Meriania aurata C. Ulloa, D. Fernández & D.A. Neill	AD145	Agnes Dellinger	Ecuador	Pastaza, Reserva EcoMinga	13.11.2016	QCNE	2	buzz-bee
Meriania costata Wurdack	AD106	Agnes Dellinger	Ecuador	Loja, PN Podocarpus	10.09.2016	WU 0092833	4	mixed-vertebrate (4)
Meriania drakei (Cogn.) Wurdack	AD132	Agnes Dellinger	Ecuador	Napo, Cosanga along roadside	14.10.2016	WU 0092810	8	buzz-bee (4)
Meriania furvanthera Wurdack	AD23	Agnes Dellinger	Ecuador	Loja, El Tiro	20.10.2012	WU 0072421	1	mixed-vertebrate (4)
Meriania furvanthera Wurdack	AD124	Agnes Dellinger	Ecuador	Loja, PN Podocarpus	13.09.2016	WU 0092838	3	
Meriania hernandoi L. Uribe	AD131	Agnes Dellinger	Ecuador	Napo, Cosanga along roadside	14.10.2016	WU 0092801	8	buzz-bee (4)
Meriania inflata Michelangeli & R. Goldenb.	RG2078	Renato Goldenberg	Brazil	Bahia	13.10.2014	NYBG02286571	1	passerine
Meriania longifolia (Naudin) Cogn.	FA10536	Frank Almeda	Colombia	Norte de Santander	25.02.2012	CAS 1127902	1	buzz-bee (5)
Meriania loxensis Gleason	AD115	Agnes Dellinger	Ecuador	Loja, PN Podocarpus	12.09.2016	QCNE	3	mixed-vertebrate
Meriania maguirei Wurdack	AD110	Agnes Dellinger	Ecuador	Loja	11.09.2016	QCNE	4	buzz-bee (4)
Meriania maxima Markgr.	MA1768	Marcela Alvear	Colombia	Narino	16.02.2013	CAS 1155921	4	buzz-bee (4)
Meriania maxima Markgr.	AD104	Agnes Dellinger	Ecuador	Pichincha, Reserva Bellavista	08.09.2016	WU 0092813	1	
Meriania mexiae Wurdack	MA1853	Marcela Alvear	Colombia	Putumayo	16.02.2013	CAS 1156500	1	buzz-bee
Meriania phlomoides (Triana)	AD78	Agnes Dellinger	Costa Rica	Heredia, Cerro Dantas	07.03.2015	WU 583185	6	mixed-vertebrate (4)
Meriania phlomoides (Triana)	AD76	Agnes Dellinger	Costa Rica	Puntarenas, Monteverde	22.02.2015	WU	4	
Meriania radula (Benth.) Triana	AD15	Agnes Dellinger	Ecuador	Loja, PN Podocarpus	11.10.2012	WU 0072420	1	mixed-vertebrate

Meriania sanguinea Wurdack	AD108	Agnes Dellinger	Ecuador	Loja, PN Podocarpus	10.09.2016	WU 0092832	8	mixed-vertebrate (4)
Meriania selvaflorensis Humberto Mend.	MA1465	Marcela Alvear	Colombia	Caldas	02.03.2011	CAS 1119760	1	buzz-bee
Meriania silverstonei Humberto Mend. & Fern. Alonso	FA10210	Frank Almeda	Colombia	Valle del Cauca	01.02.2011	CAS 1120063	1	buzz-bee
Meriania speciosa (Bonpl.) Naudin	FA10219	Frank Almeda	Colombia	Valle del Cauca	02.02.2011	CAS 1119942	1	buzz-bee
Meriania splendens Triana	MA1690	Marcela Alvear	Colombia	Narino	25.01.2013	CAS 1156411	1	buzz-bee
Meriania sp. *nov*	AD158	Agnes Dellinger	Ecuador	Pastaza, Reserva EcoMinga	15.11.2016	WU 0092855	1	buzz-bee
Meriania tomentosa (Cogn.)	AD30	Agnes Dellinger	Ecuador	Loja, PN Podocarpus	30.10.2012	WU 0072422	1	mixed-vertebrate (4, 6)
Meriania tomentosa (Cogn.)	AD105	Agnes Dellinger	Ecuador	Pichincha, Reserva Bellavista	08.09.2016	WU 0092814	2	mixed-vertebrate (4,6)
Meriania tomentosa (Cogn.)	AD111	Agnes Dellinger	Ecuador	Loja, PN Podocarpus	11.09.2016	QCNE	2	mixed-vertebrate (4, 6)
Meriania urceolata Triana	KR1446	Karen Redden	Guyana	Cuyuni-Mazaruni	08.12.2002	NY02513392	1	buzz-bee

Supplementary Table 14. Best-fit model selection for estimation of ancestral pollination syndrome using ML methods. 'ER' – equal rates model; 'ARD' – all rates different model.

model	AIC	LogL	p
ER	40.28	-19.14	
ARD	49.12	-18.56	0.95

Supplementary Table 15: Number of intact specimens and number of specimens where single landmarks were estimated, grouped by each subset within which they were estimated and into which pollination syndrome specimens are grouped.

Subset for estimation	No. intact specimens	No. estimated	Total no. subset	Pollination syndrome	Total no. syndrome
large flowered *Meriania*	12	33	45	buzz-bee	
Adelobotrys sp.	4	7	11	buzz-bee	57
Graffenrieda sp.	1	0	1	buzz-bee	
M. tomentosa group	16	12	28	mixed-vertebrate	37
M. sanguinea group	8	1	9	mixed-vertebrate	
Axinaea sp., M. inflata	33	10	43	passerine	43

Supplementary Table 16: Proportion of simulations where one or more landmarks differed significantly between the estimated and the intact set (T-test/F-test) for the four different estimation techniques. TPS was chosen to estimate landmarks in specimens with missing data.

Subset for estimation	BPCA	MS	REG	TPS
large flowered *Meriania*	0/0.03	0/0.65	0/0.01	0/0.04
Adelobotrys adscendens	0/0.22	0.01/0.38	-	0/0.15
Meriania tomentosa group	0/0.01	0/0.87	0/0.01	0/0.01
Meriania sanguinea group	0/0.13	0/0.69	0/0.31	0/0
Axinaea sp., M. inflata	0/0	0/0.8	0/0	0/0

Supplementary Literature

1. Esteve-Altava. B. In search of morphological modules: a systematic review. Biol Rev Camb Philos Soc 92(3). 1332-1347 (2017).

2. Fornoni. J. Ordano. M.. Pérez-Ishiwara. R. Boege. K. & Domínguez. C. A. A comparison of floral integration between selfing and outcrossing species: a meta-analysis. Ann Bot 117(2). 299-306 (2016).

3. Diggle. P. K. Modularity and intra-floral integration in metameric organisms: plants are more than the sum of their parts. Philos Trans R Soc Lond B Biol Sci 369(1649). 20130253 (2014).

4. Dellinger. A. S. et al. Beyond buzz-pollination – departures from an adaptive plateau lead to new pollination syndromes. New Phytol. Available at https://doi.org/10.1111/nph.15468 (12 October 2018).

5. Renner. S. S. A survey of reproductive biology in Neotropical Melastomataceae and Memecylaceae. Ann Mo Bot Gard 76(2). 496-518 (1989).

6. Vogel. S. Remarkable nectaries: structure. ecology. organophyletic perspectives I. Substitutive nectaries. Flora 192: 305–333 (1997).
