# Supplementary material for: Modularity increases rate of floral evolution and adaptive success for functionally specialized pollination systems
Source: Commun Biol. 2019 Dec 5;2:453. doi: 10.1038/s42003-019-0697-7 (PMC6895197; doi:10.1038/s42003-019-0697-7)
Supplement: Supplementary file 2 — Description of Additional Supplementary Files [file 42003_2019_697_MOESM2_ESM.pdf]

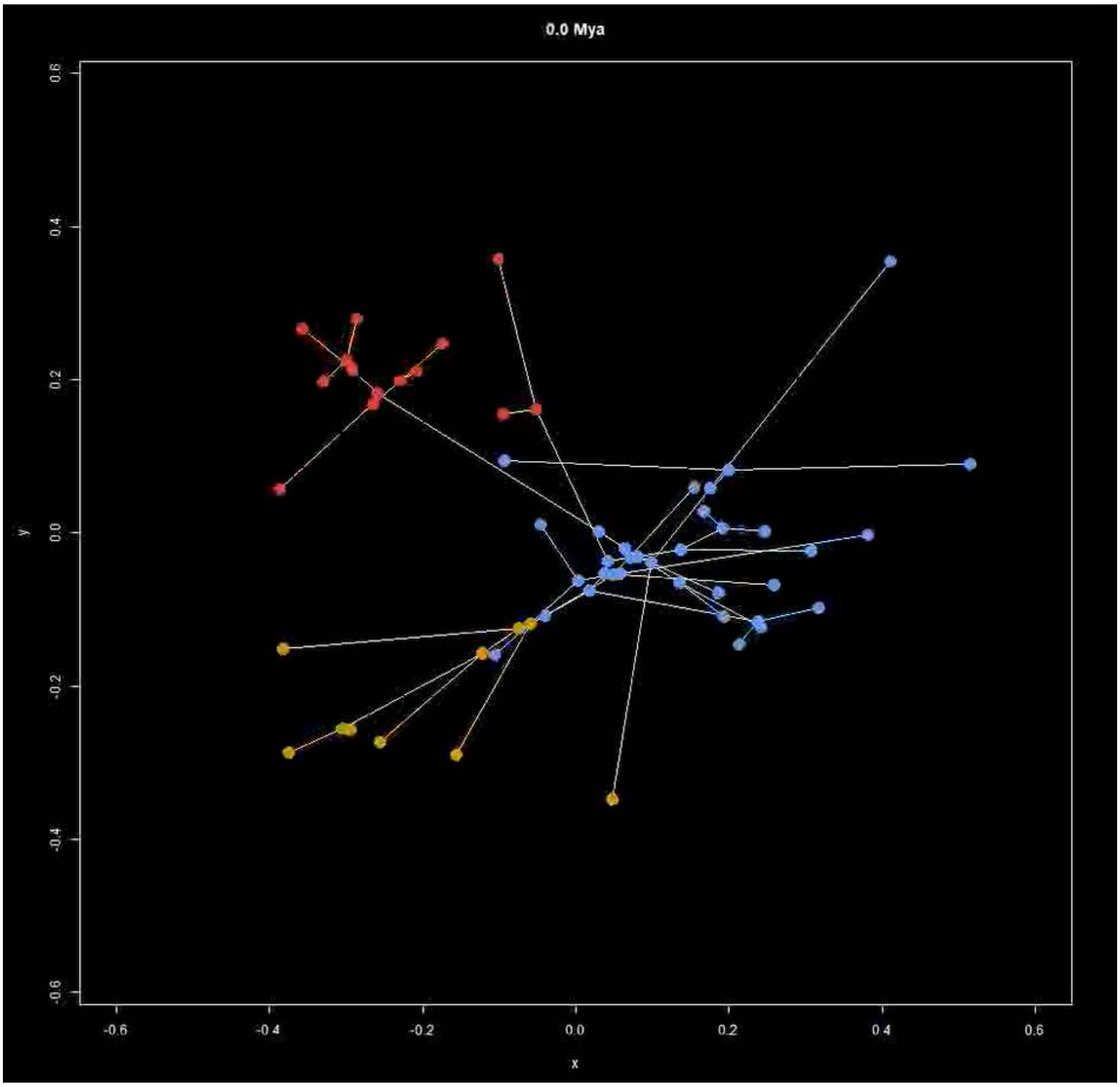

Supplementary Movie 1. Phylomorphospace of Merianieae flower shape (PC1 and PC2) through evolutionary time. Note the stasis of nodes in the central to right area of shape space occupied by extant 'buzz-bee' syndrome species ('ancestral shape space'), while branches where shifts to the 'mixed-vertebrate' or 'passerine' syndrome have occurred explore and converge in new areas of flower shape space. Branches are coloured according to reconstructions of ancestral pollination systems ('buzz-bee' – blue, 'mixed-vertebrate' – red, 'passerine' – yellow)
