# Supplementary material for: Modularity increases rate of floral evolution and adaptive success for functionally specialized pollination systems
Source: Commun Biol. 2019 Dec 5;2:453. doi: 10.1038/s42003-019-0697-7 (PMC6895197; doi:10.1038/s42003-019-0697-7)
Supplement: Supplementary file 4 — Reporting Summary [file 42003_2019_697_MOESM4_ESM.pdf]

nature research

Corresponding author(s): Agnes S. Dellinger

Last updated by author(s): Oct 2, 2019

Reporting Summary

Nature Research wishes to improve the reproducibility of the work that we publish. This form provides structure for consistency and transparency in reporting. For further information on Nature Research policies, see Authors & Referees and the Editorial Policy Checklist.

Statistics

For all statistical analyses, confirm that the following items are present in the figure legend, table legend, main text, or Methods section.

n/a	Confirmed	
☐	☒	The exact sample size (n) for each experimental group/condition, given as a discrete number and unit of measurement
☐	☒	A statement on whether measurements were taken from distinct samples or whether the same sample was measured repeatedly
☐	☒	The statistical test(s) used AND whether they are one- or two-sided<br>*Only common tests should be described solely by name; describe more complex techniques in the Methods section.*
☐	☒	A description of all covariates tested
☐	☒	A description of any assumptions or corrections, such as tests of normality and adjustment for multiple comparisons
☐	☒	A full description of the statistical parameters including central tendency (e.g. means) or other basic estimates (e.g. regression coefficient) AND variation (e.g. standard deviation) or associated estimates of uncertainty (e.g. confidence intervals)
☐	☒	For null hypothesis testing, the test statistic (e.g. F, t, r) with confidence intervals, effect sizes, degrees of freedom and P value noted<br>*Give P values as exact values whenever suitable.*
☐	☒	For Bayesian analysis, information on the choice of priors and Markov chain Monte Carlo settings
☐	☒	For hierarchical and complex designs, identification of the appropriate level for tests and full reporting of outcomes
☐	☒	Estimates of effect sizes (e.g. Cohen's d, Pearson's r), indicating how they were calculated

Our web collection on statistics for biologists contains articles on many of the points above.

Software and code

Policy information about availability of computer code

Data collection	The data used in this study was collected by ourselves using microCT-scanning (Xradia MicroXCT-200), 3D-reconstructions (XMRReconstructor SRadia Inc) and landmarking techniques on 3D models (AMIRA 5.5.0), we did not write codes for data collection. We have started making all scan data publicly available from https://phaidra.univie.ac.at/<br>The genetic data for phylogenetic analyses was produced by our group and has been published in 2019, all sequence data has been deposited on genebank (Dellinger et al. 2019, New Phytologist).
Data analysis	All morphometric data analyses were performed in the statistical computing language R (R Developmental Core Team 2018, https://www.R-project.org/) using existing and published functions implemented in the packages Geomorph (Adams et al. 2018), EMMLi (Goswami et al. 2016), Phytools (Revell et al. 2012), APE (Paradis et al. 2004), Evomap (Smaers et al. 2018), Geiger (Pennell et al. 2014), L1OU (Khabbazian et al. 2016). We will be happy to deposit codes e.g. in GitHub if this is of interest.<br>For the construction of the phylogeny, the following software was used: BEAST2 (v2.5.0), PartitionFinder 2, Tracer (v. 1.6), LogCombiner (v2.5.0) and TreeAnnotator (v2.5.0)

For manuscripts utilizing custom algorithms or software that are central to the research but not yet described in published literature, software must be made available to editors/reviewers. We strongly encourage code deposition in a community repository (e.g. GitHub). See the Nature Research guidelines for submitting code & software for further information.

Data

Policy information about availability of data

All manuscripts must include a data availability statement. This statement should provide the following information, where applicable:

- Accession codes, unique identifiers, or web links for publicly available datasets
- A list of figures that have associated raw data
- A description of any restrictions on data availability

Information on sampling details and accession numbers of all molecular sequence data analysed during this study are reported in the supplementary information files of this published article: Dellinger et al. Beyond buzz-pollination - departures from an adaptive plateau lead to new pollination syndromes. New Phytologist 221

(2) 1136-1149 (2019). https://doi.org/10.1111/nph.15468
All 3D-flower scan data are being deposited on the public data storage https://phaidra.univie.ac.at/. The landmark dataset derived from these scans is available from the corresponding author upon request.

Field-specific reporting

Please select the one below that is the best fit for your research. If you are not sure, read the appropriate sections before making your selection.

☐ Life sciences ☐ Behavioural & social sciences ☒ Ecological, evolutionary & environmental sciences

For a reference copy of the document with all sections, see nature.com/documents/nr-reporting-summary-flat.pdf

Ecological, evolutionary & environmental sciences study design

All studies must disclose on these points even when the disclosure is negative.

Study description	We studied floral shape change and changes of patterns in floral modularity with pollinator shifts in an evolutionary context. We obtained 3-dimensional models of 147 flowers of a total of 33 species by employing high-resolution x-ray computed tomography. We then placed 37 homologous landmarks on each flower model to capture its shape. Standard geometric morphometric procedures (Procrustes fitting) were used to project the flowers into shape space. We then calculated mean floral shape for each species and tested five different hypotheses of floral modularity using the R-package Geomorph. Four of these hypotheses are based on the literature and a fifth hypothesis was designed by us to better reflect trait functioning specific for our study group. We split the dataset into three groups based on the three known pollination syndromes in Merianieae and tested the modularity hypothesis separately for each pollination syndrome. To incorporate intraspecific variability, we also drew 100 random samples (without replacement) of the 147 flowers and tested modularity on each of these random samples. Finally, we tested the five modularity hypotheses on floral mean shapes across all 33 species, accounting for phylogeny using the 'phylo.modularity' function in the R-package geomorph. We then tested flower shape evolution on mean floral shape under different evolutionary scenarios (Brownian Motion, Lambda, Early Burst, Ornstein-Uhlenbeck).
Research sample	147 flowers of 33 Merianiae species, spanning the taxonomic and morphological diversity of the clade and representing the ancestral 'buzz-bee' pollination system as well as two independent shifts to a 'mixed-vertebrate' syndrome and a 'passerine' bird syndrome. 17 of the species stem from highly remote places in the South American tropical rainforests and were only represented by one specimen. Details on sampling are given in the Supplementary Material Table 1.
Sampling strategy	We aimed at sampling both across the taxonomic and morphological diversity of Merianiae and to incorporate species from the ancestral 'buzz-bee' pollination system as well as from the pollination shifts. this includes two independent shifts into a 'mixed-vertebrate' syndrome and two independent shifts into a 'passerine' syndrome. This sample is appropriate because it represents highly divergent floral phenotypes as well as species that underlie different pollinator selection regimes and hence is ideal to test contrasting hypotheses of floral evolution and convergence.
Data collection	In the field, entire anthetic flowers were collected in 70% ethanol by Agnes S. Dellinger, Diana Fernández-Fernández, Darin Penneys, Fabián Michelangeli and collaborators Frank Almeda and Marcela Alvear. Since flowers are made of soft tissue, we were careful to only include flowers which were fully anthetic to avoid any potential confounding effects due to flower age. Specifically, all flowers are buds first and then open by spreading the petals. In our study, we only used flowers which were fully open and would be visited by pollinators.<br>High-resolution X-ray computed tomography of flowers was performed by Agnes S. Dellinger and Susanne Pamperl, all scan protocols and flowers are stored at the University of Vienna and are available upon request. 3D-reconstruction of flower models was done by Agnes S. Dellinger and Silvia Artuso. The placement of the 37 homologous landmarks on the flower models was done exclusively by Silvia Artuso to avoid observer bias. Silvia Artuso and Agnes Dellinger performed an error-study to assess the quality of the chosen landmarks, i.e. whether these landmarks could be replicated. Two 3D-models were iteratively landmarked for ten times each and jointly procrustes fitted with ten additional, different specimens. In optimal landmark configurations, error in replicated samples should be close to 0 or at least one magnituede smaller than in non-replicated samples. To calculate error around each single landmark, teh mean distance of each landmark (of the 10 replicates and the 10 independent samples, respectively), was compared to the consensus. Using T- and F-tests, the mean replicate distances were compared to the mean distnaces of the non-replicates at each landmark. All landmarks placed in both replication sets were significantly less variable than in the non-replicate placements both using T- and F-tests and observer errors (mean distance of landmarks to consensus) were more than one magnitude smaller in replicates than in the non-replicate set. Thus, selected landmarks were accurate enought to proceed with further landmarking.<br>Since we wanted to provide a broad sample across Merianiae, we aimed at including as many different species as possible, i.e. as many species for which we could obtain relatively undamaged flower material. When flowers showed minor damage so that one or a maximum of 10 landmarks could not be placed, we employed advanced landmark estimation techniques which we detailed in the Suppelmentary Methods sections. Following recent recommendations by Arbour & Brown (2014), we tested four different estimation techniques by first simulating missing landmarks in our intact samples and testing whether re-estimating these landmarks would significantly alter the outcome of our study. Since there was no significant effect, we empoyed estimation techniques by the methods proposed by Arbour & Brown (2014).
Timing and spatial scale	Flowers were collected between 2011 and 2016 (one in 2002) in different field sites across South America (Supplementary Table 1). HRX-CT-scanning and landmarking was performed in March to May 2016 and in February and March 2017. Before resuming landmarking in 2017, Silvia Artuso underwent a second landmarking training by repeatedly landmarking a single specimen ten times.
Data exclusions	No flowers were excluded from the analysis.

Reproducibility	To assure accuracy of landmark placement, an error study was performed. Two 3D-models were iteratively landmarked for ten times each and jointly procrustes fitted with ten additional, different specimens. In optimal landmark configurations, error in replicated samples should be close to 0 or at least one magnituede smaller than in non-replicated samples. To calculate error around each single landmark, teh mean distance of each landmark (of the 10 replicates and the 10 independent samples, respectively), was compared to the consensus. Using T- and F-tests, the mean replicate distances were compared to the mean distnaces of the non-replicates at each landmark. All landmarks placed in both replication sets were significantly less variable than in the non-replicate placements both using T- and F-tests and observer errors (mean distance of landmarks to consensus) were more than one magnitude smaller in replicates than in the non-replicate set.
Randomization	In order to analyse the impact on floral shape by different pollinator selection regimes, we grouped flowers into 'pollination syndromes', which represent adaptations to specific functional pollinator groups. In an earlier paper (Dellinger et al. New Phytologist 2019), we tested for pollination syndromes in Merianieae by using Random Forest Analyses and could distinguish three distinct pollination syndromes, 'buzz-bee', 'mixed-vertebrate' and 'passerine'. We rigorously tested the 'mixed-vertebrate' syndrome since it is an unusual combination of different vertebrate pollinators (hummingbirds, bads, rodents, flowerpiercers). None of our analyses suggested disentangling this pollination syndrome but, on the contrary, supported treating flowers pollinated by any of these pollinator assemblages as under the same selection regime. We hence employed this syndrome classification also in this study.
Blinding	Blinding was not important in our study since landmarks have to be placed on exactly the same homologous point on each specimen. Homology is not affected by the grouping into pollination syndromes.

Did the study involve field work? ☒ Yes ☐ No

Field work, collection and transport

Field conditions	Sampling of flowers was conducted at different cloud forest field sites in South America under rainly and windy conditions.
Location	Flowers were collected at different rainforest sites in Costa Rica, Ecuador, Colombia, Brazil and Guyana, details on sampling sites and the related herbarium vouchers with detailed information on sampling localities are given in the supplementary table 1. Collections were done both in National Parks and protected areas as well as along road sites and in privates reserves. All necessary permits were obtained prior to collection (see below).
Access and import/export	All research was conducted with collection permits and export permits were obtained before exporting material. We can share all detailed permit information if required. The majority of samples were collected in Costa Rica, Ecuador and Colombia.<br>Costa Rica: permits to Agnes Dellinger by MINAE (Sistema nacional de áreas de conservación), scientific passport 05770, 25.01.2015-30.07.2015 and CONAGEBIO (R-003-2015-OT-CONAGEBIO) 25.01.2015-24.01.2018.<br>Ecuador: Contrato Marco INABIO authorizing collection of Melastomataceae to Diana Fernández-Fernández and Agnes Dellinger (MAE-DNB-CM-2016-0045), 19.07.2016 - 18.07.2019, export permits: 009-2016-IC-FLO-DNB/MA and 093-17-EXP-IC-FAU_DNB/MA<br>Colombia: project "Sistemática y filogenia de la tribu Miconieae (Melastomataceae)", Ministerio de Ambiente y Desarrollo Sostenible (MADS), permit number 19, date of issue November 9th 2010, Permit file number IDB0106-RGE0080 and Contrato de Acceso a Recursos Genéticos para Investigación Científica sin Interés Comercial No. 43, March 18th 2011, MADS<br>Brazil: INEMA/BA: 2014-008132/TEC/PESQ-0010
Disturbance	No major disturbance was caused since only a few flowers and herbarium specimens were collected from the plants, and Merianieae generally are shurbs or trees that produce hundreds of flowers. Hence, our sampling was not destructive.

Reporting for specific materials, systems and methods

We require information from authors about some types of materials, experimental systems and methods used in many studies. Here, indicate whether each material, system or method listed is relevant to your study. If you are not sure if a list item applies to your research, read the appropriate section before selecting a response.

Materials & experimental systems

n/a	Involved in the study
☒	☐ Antibodies
☒	☐ Eukaryotic cell lines
☒	☐ Palaeontology
☐	☒ Animals and other organisms
☒	☐ Human research participants
☒	☐ Clinical data

Methods

n/a	Involved in the study
☒	☐ ChIP-seq
☒	☐ Flow cytometry
☒	☐ MRI-based neuroimaging

Animals and other organisms

Policy information about studies involving animals; ARRIVE guidelines recommended for reporting animal research

Laboratory animals	The study did not involve laboratory animals.

Wild animals	In this study, pollinatin g animals were not observed. Details on pollinator observations and behaviour are found in Dellinger et al. New Phytologist 2019. We did not catch or manipulate wild animals.
Field-collected samples	Flower samples were collected from trees using scissors or picking them by hand and transferred directly into 70% alcohol for preservation. There were no experimental procedures or manipulations applied to flowers.
Ethics oversight	During fieldwork in national parks and private reserves, park authorities were informed of our collection activities. None of the species collected are of economic or pharmaceutical interest and are not protected by the CITES regulations. Hence, we are not aware of any ethical problems related to sample collection.

Note that full information on the approval of the study protocol must also be provided in the manuscript.
